# Analysis of extracellular matrix composition in the visceral muscles of *Nidogen* mutant larvae in *Drosophila*

**DOI:** 10.17912/micropub.biology.000251

**Published:** 2020-05-17

**Authors:** Uwe Töpfer, Anne Holz

**Affiliations:** 1 Technische Universität Dresden, Institute of Genetics; 2 Justus-Liebig-Universität Giessen, Institut für Allgemeine und Spezielle Zoologie, Allgemeine Zoologie und Entwicklungsbiologie

**Figure 1:**
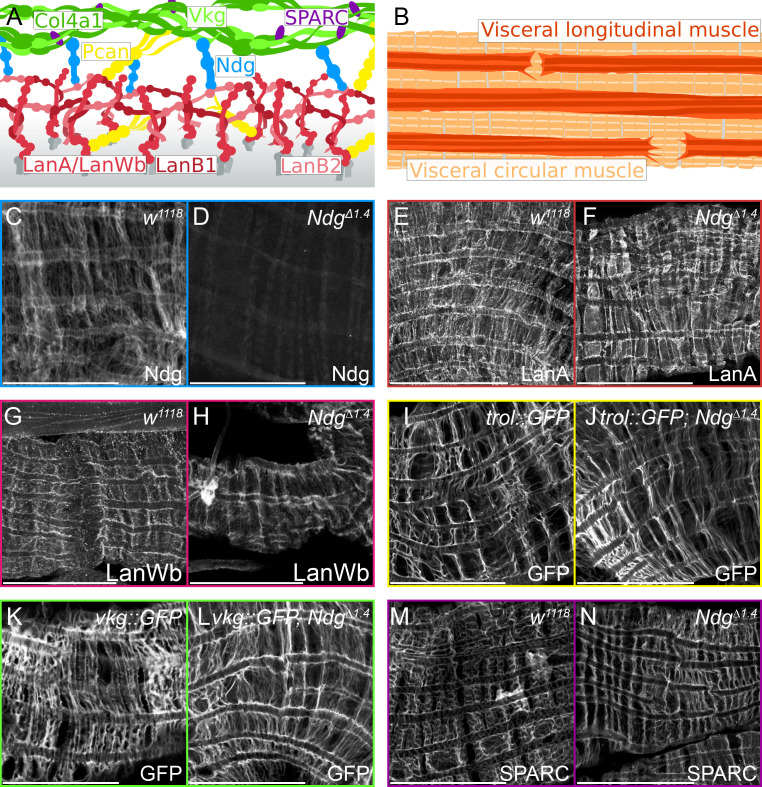
**Distribution of extracellular matrix (ECM) proteins in the visceral midgut muscles of *Ndg* mutant larvae.** (A) Schematic illustration of ECM core components. Laminin heterotrimers are anchored to cell surface receptors and are organized as a network building ternary nodes. The Laminin A and Laminin Wb heterotrimers are composed of either the α-subunit Laminin A (LanA, red) or the α-subunit Laminin Wb (LanWb, red), the β-subunit Laminin B1 (LanB1, dark red) and the γ-subunit Laminin B2 (LanB2, light red). Collateral linkage between the ECM networks is mediated by Perlecan (Pcan, yellow; encoded by the *trol* gene) as well as Nidogen (Ndg, blue), that binds to Laminin, Perlecan and Collagen IV. The Collagen IV triple helix is composed of two α1- subunits (Collagen IV α1, Col4α1, dark green) and one α2-subunit (Viking, Vkg, light green). Secreted protein, acidic, cysteine-rich (SPARC, purple) binds to the Collagen IV network. (B) Schematic illustration of the larval midgut visceral muscle morphology. Circular muscles highlighted in light orange and overlaying longitudinal muscles in dark orange. (C, D) Nidogen antibody staining in control (*white^1118^*, C) and *Ndg^Δ1.4^* mutant larvae (D). (E, F) Laminin A and (G, H) Laminin Wing blister antibody staining in control (*white^1118^*, E and G) and *Ndg^Δ1.4^* mutant larvae (F and H). (I, J) GFP antibody staining in control (*trol::GFP*, I) and *Ndg* mutant (*trol::GFP*; *Ndg^Δ1.4^*, J), (K, L) as well as in control (*vkg::GFP*, K) and *Ndg* mutant larvae (*vkg::GFP*, *Ndg^Δ1.4^*, L). (M, N) SPARC antibody staining in control (*white^1118^*, M) and *Ndg^Δ1.4^* mutant larvae (N). Scale bars = 50 µm.

## Description

A group of highly conserved extracellular matrix (ECM) proteins, which are enriched in basement membranes across all metazoa, form the so called ‘basement membrane tool kit’ (Hynes, 2012; Hynes and Zhao, 2000). These important components are organized in two layers connected to each other via the linker proteins Perlecan and Nidogen (Fig. 1A). The inner layer is formed by a self-assembling network of Laminin heterotrimers, consisting of an α-, β- and γ-subunit each. The outer network is composed of Collagen IV, which builds a triple helix with two α1-subunits and one α2-subunit. In *Drosophila melanogaster* a small set of genes encode for these components. Four genes code for Laminin subunits: two genes for α -subunits, (*Laminin A*, *LanA*, and *wing blister*, *wb*, referred to herein as ’*LanWb*’*)*, and only one gene encodes for a β- (*Laminin B1*, *LanB1*) and one gene encodes an γ-subunit (*Laminin B2*, *LanB2*). Furthermore, one gene encodes for the Collagen IV subunits α1 (*Collagen IV* α*1*, *Col4*α*1*) and α2 (*viking*, *vkg*) as well as for the linker proteins Perlecan (*terribly reduced optic lobes*, *trol*) and Nidogen (*Ndg*). Ndg connects Laminin to the Collagen IV network via binding of Perlecan and Collagen IV to the Laminin γ-subunit, and has been suggested to play an essential role in ECM assembly (Fig. 1A; Fox *et al.*, 1991; Hopf *et al.*, 2001a; Mann *et al.*, 1989; Reinhardt *et al.*, 1993). However, in contrast to mutants of other basement membrane components in *Drosophila*, *Ndg* mutants are viable and seem not to play a role in general ECM assembly (Dai *et al.*, 2018; Wolfstetter *et al.*, 2019), whereas further ultrastructural analyses reveal a disrupted ECM of larval visceral muscles (Wolfstetter *et al.*, 2019). Whether this ultrastructural alterations in the larval visceral muscles of *Ndg* mutants correspond to a change in distribution or assembly of other basement membrane components is not known, so we examine the protein distribution of the main ECM components in *Ndg* mutants in this study.

Here, we analysed the relevance of *Ndg* for ECM assembly in the visceral muscles of third instar larvae and compared basement membrane protein localization in visceral muscles of amorphic *Ndg* mutants to controls through labelling with specific antibodies and GFP exon trap lines. As expected, Ndg is evenly distributed across all visceral muscles in controls (Fig. 1C) whereas *Ndg* mutants do not show this signal (Fig. 1D), although the visceral muscles are properly formed as detected by control staining of F-actin (data not shown). The assembly of Laminin heterotrimers is essential for proper incorporation of other ECM components like Ndg (Wolfstetter and Holz, 2012; Wolfstetter *et al.*, 2019). Since visceral muscles express both possible Laminin heterotrimers and there could be some functional redundancy between them (Martin *et al.*, 1999; Wolfstetter and Holz, 2012; Yarnitzky and Volk, 1995), we studied the distribution of both Laminin α-subunits (LanA in Fig. 1E and F and LanWb in Fig. 1G and H). Congruently with the role of the Laminin network for basement membrane initiation (Hohenester and Yurchenco, 2013), loss of Ndg neither affect Laminin A nor the Wing blister heterotrimer deposition in the larval visceral muscles in general (Fig. 1E-H). The linker protein Pcan is supposed to act as collateral linker between the Laminin and the Collagen networks, additionally it is also able to bind Ndg (Battaglai *et al.*, 1992, Hopf *et al.*, 2001a, 2001b). We analysed the Pcan localisation with a *trol::GFP* exon trap line (Morin *et al.*, 2001). Comparison of GFP signals in controls and *Ndg* mutants show no obvious differences (Fig. 1I and J), suggesting that Ndg is not required to localise Pcan in the basement membrane. Localization of the Collagen IV network was studied with the *vkg::GFP* exon trap line (Morin *et al.*, 2001), which reflects the distribution of the Collagen IV triple helix. The comparable signals from controls and *Ndg* mutants (Fig. 1K and L) indicate no role of Ndg for connecting the Laminin and Collagen networks, which could be explained by a redundant function of Pcan and Ndg (Fig. 1A). This model is supported by a recent study that showed a stronger phenotype of *trol*, *Ndg* double knockdown compared to a single *trol k*nockdown (Dai *et al.*, 2018). Similar to other ECM components tested, the localization of the Collagen binding glycoprotein SPARC is unaffected in *Ndg* mutants compared to controls (Fig. 1M and N).

Our data confirm that the basic composition of the examined ECM proteins is not affected by loss of Ndg and that Ndg therefore does not appear to be the essential single player for the connection of the different basement membrane layers.

## Methods

Immunofluorescence staining

Antibody staining of *Drosophila* wandering third instar larvae was performed as described by Müller, (2008) and modified as described in Wolfstetter *et al.* (2019). The following primary antibodies were used: rabbit anti-green fluorescent protein (GFP, 1:500, Abcam, ab290), guinea pig anti-Laminin A (LanA, 1:500; Harpaz and Volk, 2012), rabbit anti-Wing blister (LanWb, 1:100; Martin *et al.*, 1999), rabbit anti-Nidogen (Ndg, 1:1.000; Wolfstetter *et al.*, 2009), rabbit anti-SPARC (SPARC, 1:500; Martinek *et al.*, 2002), Cy3 conjugated goat IgG anti-guinea pig (1:200, Dianova) and DyLight 488 conjugated goat anti-rabbit secondary antibody (1:1000; Vector Laboratories). Nonspecific binding sites were blocked with 5% goat serum. Tissues were embedded in Fluoromount G (Southern Biotech) and imaged by confocal microscopy (Leica TCS SP2) with same laser intensity for each experiment.

Fly stocks and genetics

Flies were grown under standard conditions (Ashburner, 1989) and crosses were performed at 25 °C. As control stocks we used *white^1118^* (*w^1118^*, FBal0018186), *trol::GFP* (FBal0243609)and *vkg::GFP* (FBal0191275) as exon trap lines (Morin *et al.*, 2001). For analyses in *Ndg* mutant background, *Ndg^Δ1.4^* (Wolfstetter *et al.*, 2019, FBal0346270), *trol::GFP; Ndg^Δ1.4^* and *vkg::GFP, Ndg^Δ1.4^* were used.
